# SLAM Family Receptor Signaling in Viral Infections: HIV and Beyond

**DOI:** 10.3390/vaccines7040184

**Published:** 2019-11-16

**Authors:** Patrick O’Connell, Andrea Amalfitano, Yasser A. Aldhamen

**Affiliations:** 1 Department of Microbiology and Molecular Genetics, College of Osteopathic Medicine, Michigan State University, East Lansing, MI 48824, USA, amalfit1@msu.edu (A.A.); 2 Department of Pediatrics, College of Osteopathic Medicine, Michigan State University, East Lansing, MI 48824, USA

**Keywords:** HIV, SLAM, SAP, EAT-2, SLAMF7, SLAMF6, innate immunity, adaptive immunity, immune-modulation

## Abstract

The signaling lymphocytic activation molecule (SLAM) family of receptors are expressed on the majority of immune cells. These receptors often serve as self-ligands, and play important roles in cellular communication and adhesion, thus modulating immune responses. SLAM family receptor signaling is differentially regulated in various immune cell types, with responses generally being determined by the presence or absence of two SLAM family adaptor proteins—Ewing’s sarcoma-associated transcript 2 (EAT-2) and SLAM-associated adaptor protein (SAP). In addition to serving as direct regulators of the immune system, certain SLAM family members have also been identified as direct targets for specific microbes and viruses. Here, we will discuss the known roles for these receptors in the setting of viral infection, with special emphasis placed on HIV infection. Because HIV causes such complex dysregulation of the immune system, studies of the roles for SLAM family receptors in this context are particularly exciting.

## 1. The SLAM Family of Receptors

The signaling lymphocytic activation molecule (SLAM) family of receptors are a set of nine conserved cell-surface glycoproteins present on the cell surface of immune cells (Table 1). SLAM family receptors are special in that most of them (SLAMF1, 3, 5, 6, and 7) are self ligands [1]. SLAMF2 and SLAMF4 serve as ligands for each other, and the ligands of SLAMF8 and SLAMF9 are unknown. SLAM family receptors are generally understood to function as both adhesion molecules and immuno-modulators [1]. Most SLAM family members (except SLAMF2, 8, and 9) contain immunoreceptor tyrosine-based switch motifs (ITSMs) in their cytoplasmic domain, which become phosphorylated upon receptor ligation, and serve as docking stations to recruit various SH2-domain-containing proteins. Tyrosine-phosphorylated SLAM family ITSMs recruit a number of well-characterized inhibitory phosphatases (SHP-1, SHP-2, SHIP-1), in addition to the two SLAM family unique adaptor proteins, SLAM-associated protein (SAP, also known as SH2D1A) and EAT-2 (also known as SH2D1B) [2]. 

Decades ago, it was discovered that mutations in the SAP protein, rendering it inactive, caused the rare genetic disease X-linked lymphoproliferative disorder (XLP) [3]. XLP is characterized by alterations of cellular and humoral immune responses, thus predisposing young males to Epstein–Barr virus (EBV)-associated morbidity and mortality. The discovery of this genetic association spurred research into the function of SAP, its ligands—the SLAM family of receptors—and what role these molecules play in immune regulation and response to infection. 

As SLAM receptors are present on most immune cells, in varying amounts and combinations, they comprise a complex immuno-regulatory network that plays an important role in maintaining balanced immune responses. Efforts to distill the basic elements of this system have revealed that, in general, SLAM family receptors can have activating or inhibitory functions on immune cells. Whether or not they function to promote immune cell activation typically depends on the presence of their adaptors, SAP and/or EAT-2. In the presence of these adaptors, ligation of SLAM family receptors often results in cellular activation, and in their absence, vice-versa [2]. This simple regulatory mechanism is most conserved and apparent in SLAMF7 and SLAMF4 regulated networks.

This review will focus on the specific known, and theorized roles that each of the various SLAM family members play in response to viral infection. For more detailed information on the specifics of SLAM family receptor signaling and the various adaptors capable of binding SLAM family members, please refer to any of these reviews [1,2,4,5].

## 2. Immune Responses to Viral Infections 

Host responses to viral infections comprise a complex interplay between the innate and adaptive arms of the immune system [6]. Initial responses by the innate immune system serve to quickly combat the offending pathogen and begin to prime and train the adaptive immune system to commence generation of long-lasting immunity [7]. Infection with HIV follows this paradigm, but notably, the innate immune system is not able to prevent long term infection due to some unique characteristics of HIV [8]. Here we will provide a brief summary of host anti-viral responses, using HIV as an example, and highlighting distinct ways in which HIV causes disruption of these processes, and identifying points in this process where SLAM family receptors play a role. 

Initial infection with HIV typically occurs in the vaginal or rectal mucosa, where virions infect local CD4^+^ memory T cells and macrophages. HIV infected cells eventually migrate out of the mucosa and reach draining lymph nodes where the virus is further spread to other memory CD4^+^ T cells, macrophages, and dendritic cells [9,10]. Peak viremia occurs around the third to fourth week following infection [11]. During this time the innate immune system mounts an acute anti-viral response to HIV and HIV-infected cells through the use of pattern recognition receptors (PRRs). PRRs, specifically toll-like receptors (TLRs), on macrophages, monocytes, dendritic cells (DCs), and other immune cells recognize virion glycoproteins, viral dsRNA, and viral ssRNA [12,13]. This in turn triggers signaling through NFκB, IRF3/7, and MAPK pathways among others. Signaling through these well-studied pathways produces a host of pro-inflammatory cytokines, chemokines, and viral restriction factors to help combat early infection [14]. Importantly, myeloid cell responses to PRR activation can be tuned based on signaling from SLAMF1, SLAMF5, SLAMF7, SLAMF8, and SLAMF9 [15,16,17,18,19,20,21]. Therefore, the specific microenvironment, including other immune cells interacting with PRR-responding myeloid cells, is essential for tuning myeloid cell responses by SLAMF family-dependent mechanisms. This suggests that modulating SLAM family signaling might impact host anti-viral immune responses. Indeed, we have shown previously that altering SLAM family signaling by overexpressing EAT-2 enhanced both innate and adaptive immune responses to antigens such as HIV-Gag [22]. HIV infection is notable in that it also causes breakdown of the gastrointestinal mucosal barrier allowing for translocation of bacteria and their products into the circulation [23]. Elevated levels of bacteria in the circulation trigger different TLRs contributing to broad activation of the innate immune system. Additionally, SLAM receptors (such as SLAMF1 [24], SLAMF6 [25], and SLAMF8 [20]) have also been shown to modulate innate immune responses following infection with both gram-negative and positive bacteria. Therefore, SLAM receptors may indirectly modulate HIV-induced bacterial translocation.

One of the most important anti-viral cytokines produced is IFN⍺ which is primarily produced by activated plasmacytoid dendritic cells (pDCs) [8,26,27]. pDC stimulation through TLR7/8/9 triggers production of high levels of IFN⍺ which orchestrates global anti-viral responses across nearly all cell types [28]. It has been shown recently that SLAMF9 signaling contributes to proper migration and function of pDCs [29], indicating a potential role for SLAMF9 in pDC-mediated antiviral responses. Following the acute phase of HIV infection, plasma viremia drops significantly, and circulating CD4^+^ T cell levels return to normal levels. During this time activated DCs are also able to present peptides derived from HIV-specific antigens and interact with T cells to begin to develop long-term (adaptive) immunity [7]. DC–CD8^+^ T cell interactions result in clonal expansion of virus specific memory CD8^+^ T cells, which recognize and destroy HIV infected cells presenting HIV-specific antigens [30]. SLAMF2–SLAMF4 interactions between DCs and CD8^+^ T cells, respectively, are necessary to ensure survival of DCs effectively presenting HIV-1 antigens [31]. In tandem, CD4^+^ T cells in lymph nodes activate B cells to generate HIV-specific antibody responses, a process also regulated by SLAMF5 interactions between these cell types [32,33]. For the vast majority of viral infections, this complex process is successful in fighting off the current infection and generating humoral and cell-mediated immunity (CMI) for prevention of future infections by the same pathogen. However, HIV has a number of ways to circumvent this [34,35]. 

Because the body is not able to fully clear HIV, the virus is able to create a state of chronic immune activation believed to underlie many of the secondary pathologies associated with HIV (HIV-associated neurocognitive disorder (HAND), atherosclerosis, insulin resistance, renal failure, osteoporosis, and hepatitis), and be a major cause of disease progression to AIDS [12,34]. Sustained IFN⍺ production by pDCs is implicated in this progression, a state potentially exacerbated by the presence of HIV-specific antibodies [36]. Chronic translocation of bacteria and bacterial products into the circulation from the gut, termed the “leaky gut hypothesis”, has also been suggested to contribute to these sustained responses [23]. HIV is also able to interfere with proper development of the adaptive immune response by interfering with B cell antibody production. It has been observed that gp120 can induce chronic activation of B cells through CD40-independent pathways, thus preventing the generation of powerful, broadly-neutralizing antibodies [37]. These are just a few of the many ways HIV has been shown to dysregulate the immune system; for a more detailed summary please refer to the following references [8,12].

## 3. SLAMF1 (SLAM, CD150)

The first identified member of the SLAM family, SLAMF1 was initially discovered as the binding partner of SAP [38]. It is expressed on T cells, B cells, macrophages, DCs, platelets, and hematopoietic stem cells (HSCs), and is characterized by its flexible structure and high degree of glycosylation [5]. It functions on immune cells to modulate their activation states, milieu of secreted cytokines, and phagocytic ability. With regard to phagocytosis, it has been shown that SLAMF1 is able to bind to *Escherichia coli* via OmpC and OmpF, and upon ligation, recruits Beclin-1 to its intracellular domain allowing for enhancement of bacterial phagocytosis [5].

Of all the SLAM family members, SLAMF1 has arguably been the most well-known association with viral infections, as it was discovered to be one of the receptors used by the measles virus to gain entry into cells [39]. In fact, it was later found that the measles virus binds to SLAMF1 via interactions with hemagglutinin MH-V, and that SLAMF1 is a universal receptor for all morbilliviruses (of which measles is a member). This genus-level receptor tropism likely stems from a few highly conserved domains on the extracellular region of SLAMF1 [54]. 

In addition to serving as a direct viral receptor on immune cells, SLAMF1 has various immune-modulatory roles in the immune cells it is expressed on. On cells of myeloid lineage, including macrophages and DCs, SLAMF1 is typically expressed at a low level, but becomes up-regulated in response to pro-inflammatory stimuli such as LPS, IL-1β, TNFα, and IL-6 [5,15]. The precise function of SLAMF1 on activated DCs has been debated in the literature. Bleharski et al. initially described SLAMF1 activation on CD40L-stimulated DCs as being pro-inflammatory, noting increased levels of IL-8 and IL-12 [16]. However, a later paper by Rethi et al. demonstrated that SLAMF1 signaling on CD40L-stimulated DCs was in fact inhibitory, with decreased IL-12 secretion and impaired ability to induce naïve T cells into Th1 cells [15]. The difference in results was attributed to the mode of SLAMF1 activation, with the initial report using soluble anti-SLAMF1 antibodies, and the latter paper using L929 cells stably expressing SLAMF1 as the mode of receptor ligation [15]. The discrepancy in the results obtained using these different methods suggests that the first report, using a soluble anti-SLAMF1 antibody, may be blocking the SLAMF1 receptor, as most soluble antibodies perform blocking functions unless they have been specifically designed as agonistic antibodies.

Complicating matters further is a study demonstrating that SLAMF1 knockout macrophages have impaired production of IL-12p70, IL-12p40, TNF⍺, and nitric oxide [17], suggesting SLAMF1 may be an activating receptor. It is hard to compare this study to the two previously mentioned studies, as those were performed using primary human cells and this study used a murine model. Differences in immune responses to signaling from various members of the SLAM family has been noted between human and murine immune cells [18,44].

SLAMF1 has also been described to have a specific role in T cells. SLAMF1 was noted to be necessary for optimal production of IL-4 and IL-13, classical Th2 polarizing cytokines [17,40]. This suggests that SLAMF1 plays a role in regulating the production of antibodies during infections. Altogether, the role of SLAMF1 in infection is interesting in that it both acts directly as a viral cell surface receptor, and modulates signaling on immune cells key to combating viral and bacterial infections.

## 4. SLAMF2 (CD48)

SLAMF2 is unique among SLAM family members in that it is expressed on most lymphocytes, contains a Glycosylphosphatidylinositol (GPI) anchor, and is not a homotypic receptor. SLAMF2 is the ligand for SLAMF4 (2B4), and vice-versa. Additionally, in rodents, SLAMF2 is also able to bind CD2, but this binding is not conserved in humans which can make translating discoveries in murine models regarding SLAMF2 function difficult [55]. On immune cells it has been observed to generally function as an adhesion molecule and co-stimulator of NK and T cells. 

**Figure 1:**
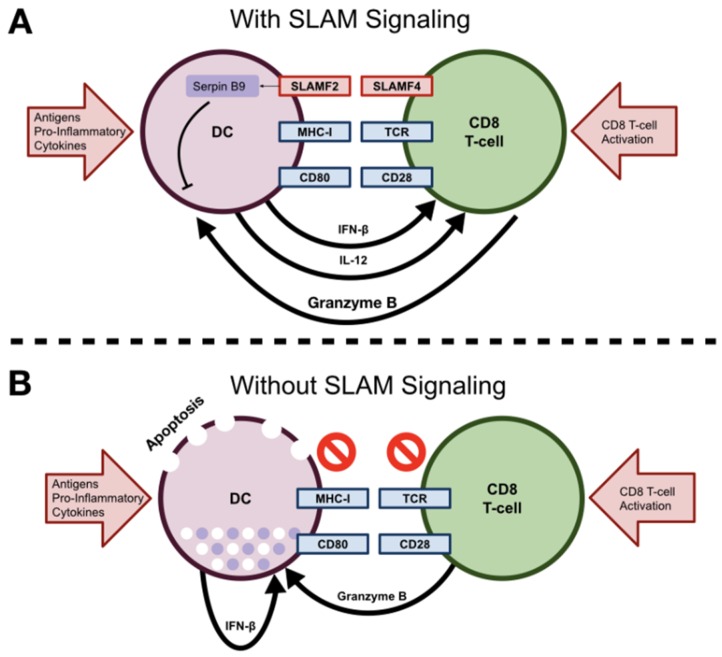
Role of SLAMF2 in orchestrating DC–T cell interactions. (**A**) Ligation of SLAMF2 on DCs (or other antigen presenting cells) via interactions with SLAMF4 on an interacting CD8 T cell results in increased expression of SERPINB9. SERPINB9 protects DCs from lysis granzyme B-dependent T cell lysis. (**B**) Interactions between DCs and CD8 T cells when SLAMF2–SLAMF4 interactions are absent on DCs, which renders DCs susceptible to apoptosis, both from autocrine IFNβ production and by granzyme B produced by CD8 T cells.

SLAMF2 was initially believed to play an indirect role in regulating CMI during viral infections, as SLAMF4 is a well described modulator of NK and CD8^+^ T responses during viral infections [31,56,57,58]. In support of this indirect role it was shown that SLAMF2 ligation on free DNA-activated DCs (mimics viral infection) prolonged their survival [31]. The mechanism underlying this effect was shown to be due to decreased apoptosis via impaired production of DC-generated IFNβ [31]. Furthermore, SLAMF2 ligation on activated DCs induced the production of granzyme B inhibitor protease inhibitor-9 (SERPINB9), which prevented T cell-mediated lysis of DCs (Figure 1A) [31]. This led to the proposition of an intriguing model where proper SLAMF2–SLAMF4 interaction between DCs and activated T cells (express high levels of SLAMF4) allows for enhanced DC survival and antigen presentation to assist in clearing infections. In scenarios where there was an absence of CD8^+^ T cell–DC interactions (i.e., no SLAMF2–SLAMF4 interaction), the DC undergoes apoptosis via production of its own pro-inflammatory cytokine, IFNβ (Figure 1B). This regulatory mechanism helps to ensure that only DCs presenting useful antigens to CD8^+^ T cells persist, and other DCs that are inducing non-specific, background inflammation do not persist [31]. This complex model describing SLAM family receptor interactions between various immune cells in the context of viral infection shows how SLAM family receptors are important maestros orchestrating immune responses.

Furthermore, SLAMF2 has been identified as being a direct modulator of CD4^+^ T cell and macrophage inflammatory responses [59,60]. Studies utilizing SLAMF2 deficient mice revealed that SLAMF2 is necessary for proper CD4^+^ T cell activation and proliferation [59,60]. Additionally, it was determined that SLAMF2 expression on macrophages was necessary for optimal TNF⍺ and IL-12 production in response to innate stimuli [60]. 

**Figure 2:**
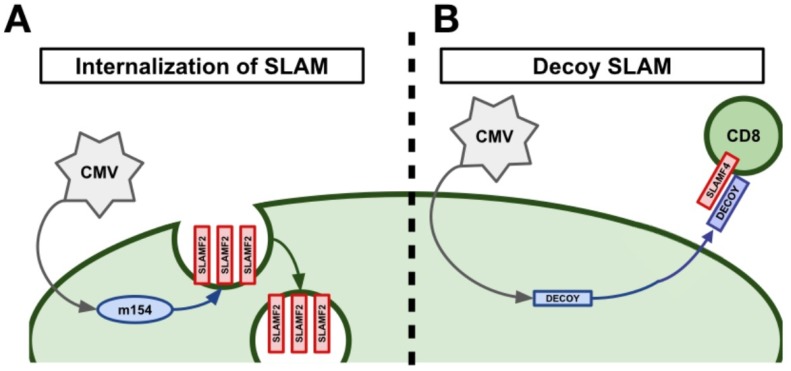
Viral manipulation of SLAMF2 to evade host immunity. (**A**) Following infection, the CMV viral protein m154 proteolytically degrades SLAMF2 in order to decrease receptor expression on the cell surface. (**B**) Owl Monkey CMV produces a decoy SLAMF2 receptor following infection of a cell. The SLAMF2 decoy protein is secreted and, once soluble, prevents proper SLAMF2–SLAMF4 interactions from occurring between CMV infected cells and cytotoxic lymphocytes, respectively.

Due to SLAMF2’s importance in regulating CMI, it was theorized that some viruses may attempt to modulate SLAMF2 to circumvent its role in combating viral infections. Indeed, it was found that cytomegalovirus (CMV), HIV, and potentially, human T cell leukemia virus type 1 (HTLV-1), have evolved to do just this [41,43,57,58]. CMV is able to evade SLAMF2-mediated immune responses via two independent mechanisms: down-regulation of SLAMF2 on infected cells via the viral protein m154 [41] and by producing a soluble SLAMF2 decoy receptor [43]. The CMV viral protein m154 was shown to directly down-regulate SLAMF2 in infected macrophages via proteolytic degradation (Figure 2A) [41]. This led to impaired NK cell cytotoxicity against CMV infected cells and, critically, infection with a m154 deficient CMV virus displayed an attenuated phenotype in vivo [41]. Certain strains of CMV, notably Owl Monkey CMV, encode a decoy SLAMF2 protein in their genome [43]. Infection of cells with these viruses was observed to secret high levels of a soluble SLAMF2 decoy protein [43]. These SLAMF2 decoy proteins were able to bind with high affinity to SLAMF4 on host immune cells and could prevent NK cell adhesion and cytotoxicity against infected cells (Figure 2B) [43]. A similar, but less well understood, method of SLAMF2 modulation occurs during HIV infection [57]. In vitro cell culture experiments using primary human T cells and HIV-1 showed that HIV-1 infected T cells down-regulated a number of important NK cell ligands including SLAMF2 [57]. This prevented optimal NK cell-mediated lysis of HIV-1 infected T cells [57]. Additionally, there is preliminary evidence that the tax protein from HTLV-1 is able to down-regulate SLAMF2 in infected cells and prevent CD8^+^ T cell-mediated cytotoxicity [58]. 

However, not all viruses display the ability to interfere with host immunity through SLAMF2-specific mechanisms. Epstein–Barr virus (EBV) infection of B cells is known to induce up-regulation of SLAMF2, which has been suggested to assist in CMI towards infected cells [56]. Such a mechanism would in theory allow for better adhesion and cytotoxicity (via SLAMF4 signaling on CD8^+^ T cells and NK cells). It is clear that SLAMF2 plays an important role in controlling cell-mediated immunity in the setting of viral infections, and we are only just beginning to understand the breadth of evasion strategies used by viruses to undermine it 

## 5. SLAMF3 (Ly-9, CD229)

The third member of the SLAM family of receptors, SLAMF3, is widely expressed on lymphocytes, notably on CD4^+^ T cells, CD8^+^ T cells, B cells, plasma cells, NK cells, NKT cells, innate lymphoid cells (ILCs), DCs, monocytes, macrophages, and hematopoietic stem cells (HSCs). Interestingly, it is also known to be expressed on hepatocytes [42]. SLAMF3 is a relatively understudied member of the SLAM family, and to date, studies have only revealed roles for SLAMF3 in control of T cell inflammatory responses and NKT cell development [5]. 

SLAMF3 may play a role in adenovirus (Ad) infections, as a large-scale interactome study using the E3 protein from Ad revealed binding to SLAMF3, in addition to a number of other SLAM family members [61]. The importance and consequences of this interaction are not yet known and this represents an interesting area of future study. 

Compared to other SLAM family members, many of which modulate DC and macrophage function in some manner, loss of SLAMF3 on macrophages was not observed to affect immune responses [62]. While loss of SLAMF3 on T cells was shown to impair Th2 responses [62], mice with global loss of SLAMF3 did not display enhanced susceptibility to LCMV infection in the same manner as SAP knockout mice do [62]. This suggest that while SLAMF3 does play some role in modulating immune cell function, the effects are not as important as that of other SLAM family members and may be redundant. 

SLAMF3’s expression on hepatocytes is its only known direct link to viral infections. A single report has shown that HCV is able to bind directly to the first N-terminal domain of SLAMF3 and use this as a cellular entry mechanism [42]. More specifically, they noted that the HCV envelope protein, E2, was responsible for this interaction. In further support of this important interaction for HCV pathogenesis, it was observed that SLAMF3 expression on hepatocytes correlated with their susceptibility to HCV infection [42]. 

## 6. SLAMF4 (2B4, CD244)

SLAMF4 is arguably the most well studied member of the SLAM family of receptors, with extensively-characterized functions on multiple lymphoid cell types [63]. SLAMF4 is expressed on NK cells, CD8^+^ T cells, NKT cells, γδ T cells, monocytes, basophils, eosinophils, DCs, and mast cells [1,5]. Deciphering the underlying signaling mechanisms of SLAMF4 have been complicated by the fact that it can bind both SAP and EAT-2 adaptors, in addition to numerous inhibitory phosphatases. Furthermore, studies have shown that the antigen density of SLAMF4 on CD8^+^ T cells differentially determines its function, with ligation of SLAMF4 when it is present at low levels inducting activating signals, and ligation of SLAMF4 when it is present at high levels producing inhibitory signals [45,64,65]. A further difference between SLAMF4 and other SLAM family members is that it is not a homotypic receptor and functions via ligation to SLAMF2.

To data, SLAMF4 is best known for its function on CD8^+^ T cells as an exhaustion marker and inhibitory receptor. This fact, combined with our current knowledge that chronic viral infections induce large numbers of exhausted CD8^+^ T cells, has contributed to a significant body of knowledge regarding SLAMF4 in the setting of chronic viral infections. Studies of SLAMF4 on CD8^+^ T cells of patients with chronic hepatitis B virus (HBV) infection have shown that chronic HBV infection increases the number of SLAMF4^+^ CD8^+^ T cells both in the peripheral circulation and in the liver, often with co-expression of PD-1 [46]. Clearance of viral infection decreased the numbers of SLAMF4^+^ CD8^+^ T cells, and blocking of SLAMF4 ex vivo allowed for improved T cell proliferation, cytokine production, and cytotoxicity [46]. Very similar effects were noted in patients with chronic hepatitis C virus (HCV) infection [47]. In the setting of chronic HCV infection, SLAMF4 was observed to be up-regulated on virus-specific CD8^+^ T cells, functioned as an inhibitory receptor on these cells, and prevented virus-specific CD8^+^ T cell proliferation [47]. A separate report, studying the role of SLAMF4 in HIV infection, noted that respiratory syncytial virus (RSV)-specific CD8^+^ T cells in HIV^+^ patients responded differentially to SLAMF4 ligation. The subset of RSV-specific CD8^+^ T cells that had the capability to down-regulate SLAMF4 were able to respond best to stimulation and those that retained high levels of SLAMF4 showed impaired IFNγ production [48]. 

The role of SLAMF4 directly on HIV-specific CD8^+^ T cells has also been studied [66]. During HIV infection, patients with elevated SLAMF4^+^ CD8^+^ T cells showed marked disease progression in comparison to those with low levels of SLAMF4^+^ CD8^+^ T cells [66]. This suggested that SLAMF4 may be a marker of dysfunctional CD8^+^ T cells or activated CD8^+^ T cells that preferentially destroy host CD4^+^ T cells; both of which could lead to disease progression in HIV. Support for the former hypothesis came from a study looking at SLAMF4 signaling more in-depth [49]. Here it was observed that HIV-1-specific CD8^+^ T cells from patients exhibited an immature phenotype, displaying high levels of SLAMF4 and PD-1. Co-expression of these two markers correlated with other markers of disease progression including viral load. Blocking of SLAMF4 and PD-1 in ex vivo T cell cultures from HIV patients allowed for increased HIV-1-specific CD8^+^ T cell proliferation [49]. This suggested that SLAMF4 expression on CD8^+^ T cells in the setting of chronic HIV-1 infection is a true inhibitory receptor. Confirming this, is work showing that SLAMF4^+^ CD8^+^ T cells from HIV-1 negative persons, stimulated with HIV-antigen-pulsed DCs, have less cytolytic activity compared to SLAMF4^-^ CD8^+^ T cells [50]. 

In contrast to the number of reports studying the inhibitory function of SLAMF4 on CD8^+^ T cells, there are a few reports describing activating effects for SLAMF4 on CD8^+^ T cells during viral infections [44,67]. For example, it has been shown that HIV elite controllers harbor a population of HIV-specific SLAMF4^+^ CD8^+^ T cells with high cytolytic capacity, and this unique subset is not commonly present in chronic progressors [68], suggesting that signaling downstream of SLAMF4 might be important for the control of HIV infection. In the setting of HTLV-1 infection, which can present with a CD8^+^ T cell-mediated encephalitis, elevated levels of SLAMF4 were observed on CD8^+^ T cells compared to uninfected controls [67]. Additionally, infected individuals presenting with neurological disease had elevated levels of SAP in their CD8^+^ T cells compared to those without neurological disease. Blockade of SLAMF4 signaling in ex vivo cultures inhibited production of cytotoxic factors, suggesting that, in this setting, SLAMF4 is functioning as an activating receptor. It is possible that the high levels of SAP, which are known to control SLAMF4 signaling, are responsible for SLAMF4 functioning as an activating receptor here. Similarly, there is evidence that SLAMF4 plays an activating role during influenza infection [44]. Here, SLAMF4 was discovered to be able to directly bind the influenza viral HA protein, stimulate CD8^+^ T cell activation, and induce lysis of influenza-infected cells. This activating effect could potentially be attributed to the binding of SLAMF4 to a different ligand than it normally does. Interestingly, the authors found that murine SLAMF4 lacked the conserved residues in human SLAMF4 that were required for binding to influenza HA, and as expected, could not bind to influenza [44]. This further highlights the complexities of studying SLAM family members using murine models.

While SLAMF4 has been best studied on CD8^+^ T cells, there are a number of reports assessing the role of SLAMF4 on NK and NKT cells in the setting of HIV infection [69,70]. In NK cells it was found that SLAMF4 expression increased on NK cells over a course of two years on HIV^+^ individuals, even in the presence of combined anti-retroviral therapy (cART) [69]. Additionally, reductions in the number of SLAMF4^+^ NK cells negatively correlated with levels of cells containing pro-virus [69]. Together, this suggests that SLAMF4 signaling on NK cells in the setting of chronic HIV infection is important for viral control via CMI. Invariant NKT cells (iNKT) are an understudied subset of NKT cells which play a role in innate immunity. A recent study looking into the role of SLAMF4 on iNKT cells during chronic HIV infection found potentially important associations [70]. It was observed that iNKT cells from HIV^+^ individuals have high levels of SLAMF4 on their CD4^+^ iNKT cells and show impaired IFNγ production. Furthermore, the percent of SLAMF4^+^ iNKT cells inversely correlated with CD4^+^ T cell count and CD4^+^/CD8^+^ ratio, and positively correlated with viral load [70]. This indicates that dysregulation of SLAMF4 on iNKT cells may be a marker of disease progression, and potentially, excessive CMI, causing destruction of host lymphocytes. While the precise roles of SLAMF4 signaling both in a variety of immune cell types and a variety of viral infections remains to be determined, it is clear that HIV and other viral infections up-regulate SLAMF4 and that this is a potential mechanism underlying immune dysfunction.

## 7. SLAMF5 (CD84)

SLAMF5 is broadly expressed on many immune cell types including B cells, T cells, monocytes, macrophages, DCs, platelets, thymocytes, NK cells, NKT cells, basophils, and eosinophils. SLAMF5 can bind to both SAP and EAT-2, and is best known for its role in regulating adhesion between T cells and B cells, allowing for proper humoral responses, it also plays a role in regulating immune signaling in numerous other immune cells. 

In contrast to some of the other SLAM family receptors, the only known association between SLAMF5 and viral infections is from a large-scale interactome screen [61]. Similarly to SLAMF3, SLAMF5 was observed to bind to E3 from adenovirus with high affinity. Follow-up studies discovered that binding of E3 proteins to SLAMF5 stimulated activation of the receptor as evidenced by phosphorylation of its ITSM. This receptor activation had functional importance as it inhibited T cell receptor signaling in Jurkat cells as determined by ERK1/2 phosphorylation [61]. While these in vitro studies of SLAMF5 signaling in the context of T cell activation in adenovirus infection are interesting, simple studies using SLAMF5 deficient mice in the context of adenovirus infection or Ad-mediated vaccination strategies [22,71,72] would be important to confirm physiologic significance. 

Dendritic cell responses to viral infections are important for generating both innate and adaptive anti-viral responses. SLAMF5 signaling in DCs is necessary for proper pro-inflammatory immune responses involving IL-1β, IL-23, and IL-12, and this is dependent on IRF8 [19]. Specifically, IRF8 is induced in DCs when they become activated and is an important transcription factor that orchestrates many of their responses [73]. Agod et al. have shown that SLAMF5 signaling in DCs is important for stabilizing IRF8, preventing its degradation by TRIM21, and allowing for proper inflammatory responses [19]. 

While T cell–DC interactions are important in viral immune responses, T cell–B cell interactions are critical for developing long-term humoral immunity; SLAMF5 has been described as being vital for this interaction [74]. Adhesion via SLAMF5-SLAMF5 homotypic interactions between B cells and T cells allows for appropriate germinal center formation in vivo [74]. This interaction requires the SLAM family adaptor SAP, and also interactions involving SLAMF6. Further supporting this is a study showing that SLAMF5 is up-regulated on memory B cells, is able to recruit both SAP and EAT-2, and functionally signals in memory B cells [75]. Furthermore, in a murine model of Systemic Lupus Erythematosus (SLE), SLAMF5 was shown to be important in preventing spontaneous germinal center formation and auto-antibody production, characteristic pathologies found in humans with SLE [33]. Proper regulation of humoral immunity is important in a number of viral infections, notably HIV-1, where it has been shown that the body has the potential to develop potent broadly neutralizing antibodies against HIV-1 [76]. Importantly, the development of these broadly neutralizing antibodies can be a double-edged sword, as anti-HIV-1 antibodies can also chronically activate pDCs and allow for sustained IFN⍺ production even in the presence of viral suppression [36]. Studies of SLAMF5 signaling in this pathogenic immune response would be highly relevant to understanding the body’s natural response to HIV-1. 

An additional manifestation in chronic HIV-1 infection is the phenomenon of T cell exhaustion, and similar to SLAMF4, SLAMF5 has been implicated as playing a role in this process [77]. In a chronic lymphocytic leukemia (CLL) model of T cell exhaustion, Lewinsky et al. observed that SLAMF5 interactions between T cells and stromal cells in their microenvironment are able to induce PD-L1 up-regulation throughout the microenvironment [77]. This in turn contributes to increased PD-1 expression on CD8^+^ T cells and results in T cell exhaustion. Studies assessing this same mechanism in the setting of chronic viral infection, such as HIV-1, are a promising area of future research.

## 8. SLAMF6 (NTB-A, Ly-108, CD352)

SLAMF6 is a particularly interesting SLAM family member to study in the context of viral infection, as it has both potent immuno-modulatory functions on cells important for CMI, and has a well-described interaction with the HIV-1 virus. SLAMF6 is expressed on NK cells, NKT cells, T cells, B cells, macrophages, pDCs, DCs, thymocytes, eosinophils, and neutrophils. SLAMF6 facilitates immune cell adhesion and also regulates immune cell signaling in a manner similar to other SLAM family members. 

SLAMF6 is the only known SLAM family member to have direct interaction with HIV-1, which occurs through the viral protein vpu [51,52,53]. Initial studies found that SLAMF6–SLAMF6 interactions between infected T cells and NK cells were important for NK cell-mediated lysis of infected cells [51]. The vpu protein from the HIV-1 genome was observed to specifically down-regulate SLAMF6 on infect cells and this prevented efficient NK cell-mediated lysis [51]. Biochemical studies revealed that vpu bound to SLAMF6 through its transmembrane domain and induced proteolytic degradation. Follow-up studies by a separate group determined that an additional mechanism through which vpu is able to down-modulate SLAMF6 is via alterations in SLAMF6 glycosylation which affected its anterograde transport [53]. The alterations in SLAMF6 glycosylation resulted in the formation of a more immature form of SLAMF6, which was preferentially sequestered in the Golgi apparatus. Of note, there is a single study contradicting the ability of vpu to down-regulate SLAMF6 on infected cells [52], but it should be noted that results obtained from this study might require further investigation, as they used an early murine model of in vivo HIV-1 infection which is now known to have limitations [78,79]. Additionally, there are important differences between murine and human SLAM family receptors that could be playing a role here. An interesting point to consider is that all of these studies looked at NK cell-CD4^+^ T cell SLAMF6 interactions, but never myeloid-CD4^+^ T cell interactions. As SLAMF6 is also present on myeloid cells [5], it is interesting to consider how SLAMF6 on myeloid cells affects HIV infected monocytes.

SLAMF6 has also been identified as a modulator, or potential modulator, of CD8^+^ T cell and NK cell function. A recent study identified SLAMF6 as a marker of progenitor exhausted CD8^+^ T cells [80]. This is particularly noteworthy, as the progenitor subset of exhausted cells, in contrast to terminally exhausted cells, are capable of reversing their exhaustion in response to PD-1 blockade [80]. While this study merely used SLAMF6 as a marker of progenitor exhausted CD8^+^ T cells, it remains to be seen what, if any role, SLAMF6 has functionally in this important immune cell type. Furthermore, this study used a tumor model of T cell exhaustion and it is not known whether or not SLAMF6 expression will mark progenitor exhausted CD8^+^ T cells in a viral model of T cell exhaustion. Finally, SLAMF6 also has a described role for regulating NK cell-mediated lysis of influenza infected cells, similar to that described above for SLAMF4 [44].

## 9. SLAMF7 (CRACC, CS1, CD319)

The seventh member of the SLAM family, SLAMF7, is increasingly being recognized as both a marker and modulator of inflammation in a number of diseases. SLAMF7 is expressed on NK cells, CD4^+^ T cells (low levels), CD8^+^ T cells, NKT cells, classical monocytes (low levels), inflammatory monocytes (high levels), macrophages, DCs, B cells, and plasma cells. It is notably absent on granulocytes [1,18]. SLAMF7 signaling is less complex than other SLAM family members in that its ITSM can only bind to EAT-2, and not SAP In the presence of EAT-2 it performs functions that can generally be described as activating, and in the absence of EAT-2 it recruits a number of inhibitory phosphates (SHP-1, SHP-2, SHIP-1, CD45, and csk) to inhibit cellular functions [81,82,83]. However, there may be yet other, unidentified mediators of SLAMF7’s inhibitory signaling, as we and others have found [18,84]. Likewise, the positive, EAT-2-mediated signaling mechanism from SLAMF7 is not entirely elucidated, but it is known that EAT-2 can signal via PLCγ1, PLCγ2, ERK1/2, and PI3K to induce Ca^2+^ flux in NK cells [82]. 

**Figure 3:**
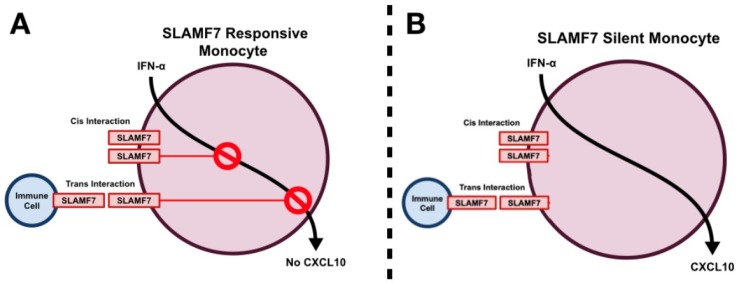
SLAMF7 silent and responsive monocytes. SLAMF7, which is present at low levels on monocytes and upregulated in response to IFN⍺, typically restrains production of alpha chemokines such as CXCL10 (**A**). This can occur either through cis interactions (ligation of SLAMF7 receptors found on the same cell) or trans interactions (ligation of SLAMF7 receptors found on separate cells (**A**). In a subset of HIV^+^ individuals SLAMF7 signaling on monocytes is unable to inhibit production of CXCL10, resulting in elevated levels of peripheral immune activation (**B**).

While no studies have yet described a direct interaction between any particular virus and SLAMF7, there are numerous reports describing immuno-modulatory roles for SLAMF7 in the setting of viral infections. Our group has recently identified SLAMF7 as playing an important role in the modulation of peripheral immune activation in chronic HIV-1 infection [18]. We showed, using a cohort of middle-aged cART-treated HIV patients, that there are elevated numbers of SLAMF7^+^ cells in HIV patients compared to controls, and that patients who do not respond to therapy have severely elevated levels of SLAMF7. More in-depth analyses revealed that CD16^+^ monocytes were responsible for this increase in SLAMF7^+^ cells which fits with previous studies showing CD16^+^ monocytes are increased in chronic HIV-1 infection [27,85,86]. In vitro studies using samples from HIV-1 patients identified SLAMF7 as being a potent inhibitor of specific type I interferon-induced chemokines (CXCL9, CXCL10, CXCL11, and CXCL12) produced by monocytes (Figure 3A) [18]. Interestingly, this inhibitory mechanism was not retained in a subset of HIV-1 individuals termed SLAMF7-silent (SF7S), and these individuals all had high levels of multiple inflammatory cytokines and chemokines in their plasma (Figure 3B). Finally, in vitro HIV-1 infection studies revealed that SLAMF7 activation on monocytes could robustly prevent their infection with HIV-1 through a CCR5/CCL3L1 mechanism [18]. Together, this work highlights the important role that SLAMF7 plays in modulating immune responses in the setting of chronic HIV infection.

SLAMF7 has also been described to have an important role in a poly I:C/D-galactosamine model of hepatitis [87]. Poly I:C is a mimic of dsRNA and simulates viral infection; its use in combination with D-galactosamine has been shown to mimic fulminant hepatitis [88]. Here it was discovered that poly I:C induced SLAMF7 expression on hepatic NK cells, as well as kupffer cells [87]. Blocking the SLAMF7–SLAMF7 interactions between hepatic NK cells and kupffer cells decreased the severity of liver injury and decreased IFNγ and TNFα levels in the liver [87]. This study suggests that excessive SLAMF7 signaling between NK cells and SLAMF7 expressing cells in their microenvironment can cause pathological inflammation, highlighting the importance of SLAMF7–SLAMF7 interactions between various immune cells during viral infection. As chronic HIV-1 infection is known to up-regulate SLAMF7 on monocytes and T cells [18], it is interesting to hypothesize how this affects other SLAMF7 expressing immune cells which regularly interact with monocytes, and what effect this would have on chronic inflammation in HIV infection.

## 10. SLAMF8 (BLAME, CD353)

SLAMF8, and its close relative, SLAMF9 are the most understudied members of the SLAM family. This is in part due to their relatively recent discovery, as well as the fact that they do not have ITSMs in their cytoplasmic domain [89,90]. Because of this, it was theorized that these receptors may not signal; however, they do contain a short cytoplasmic domain with a very high number of positively charge amino acids, suggesting a potential for protein–protein interactions and downstream signaling [89,90]. SLAMF8 is expressed on B cells, CNS macrophages, hepatic macrophages, monocytes, CD8^+^ T cells, DCs, and neutrophils. Murine microglia, a macrophage-like cell located in the brain parenchyma, have been noted to express SLAMF8 at the mRNA level, but have not yet been confirmed to express SLAMF8 at the protein level on the cell surface [91].

Our current knowledge of SLAMF8 suggests it is sensitive to innate immune stimuli and has an immuno-regulatory role in macrophages; factors that suggest it may also be involved in immune regulation during viral infection. Notably, SLAMF8 was observed to be up-regulated at the mRNA level in brain samples from patients co-infected with HIV-1 and mycobacterium tuberculosis (MTB) [92]. Since myeloid cells in the CNS (macrophages, monocytes, and microglia) are most likely to express SLAMF8, it is likely that these cells have upregulated the expression of this receptor in response to chronic CNS inflammation associated with HIV-1 and/or MTB infections. 

Insight into what role SLAMF8 could potentially be playing in myeloid cells of the CNS during viral infection comes from a report utilizing SLAMF8 knockout mice [20]. Here, the authors observed that SLAMF8 served as a negative regulator of Nox2 activity in macrophages in response to innate or bacterial challenge. This suggests that SLAMF8 up-regulation in the setting of CNS infection may be a natural anti-inflammatory response. It also suggests that SLAMF8 is able to signal in some manner, even though it lacks an ITSM. However, more recently, another report has been published with conflicting results [93]. In this SLAMF8 knockout mouse model, the authors do not note any differential response to inflammatory stimuli. Two explanations for this discrepancy are that 1) the mice used in each study are on different backgrounds (Balb/C in [20] and C57BL/6 in [93]) and 2) the readouts assessed in each report are different. Interestingly, in the C57BL/6 SLAMF8 knockout model, when both SLAMF8 and SLAMF9 were knocked out the authors observed decreased macrophage activation, suggesting they may have redundant functions as activating receptors in macrophages [93]. Interestingly, it was found that SLAMF8 and SLAMF9 were required to maintain normal levels of TLR4 expression [93]. This may have important implications in chronic HIV infection, where it is known that many patients suffer from a “leaky gut”, allowing egress of bacteria into circulation, which can activate the immune system. These data suggest that SLAMF8 and SLAMF9 may play a role in this pathological process. 

An additional role for SLAMF8 in the regulation of phagocyte motility has also been described [94]. SLAMF8 was observed to inhibit macrophage migration in a manner opposite to that of SLAMF1 [94]. This is noteworthy, as phagocyte migration to lymph nodes, the CNS, and other locations during acute HIV and other viral infections is critically important to beginning to mount an immune response against the pathogen. This migration can actually have detrimental effects in HIV where monocytes and other phagocytes, become infected early in the disease course and migrate to the CNS where they set up a viral reservoir [95,96,97]. Future studies to understand the role of SLAMF8 signaling in early HIV-1 infection are certainly warranted.

## 11. SLAMF9 (SF2001, CD84H1)

Similar to SLAMF8, SLAMF9 is a newly discovered member of the SLAM family of receptors. SLAMF9 is the most recently identified SLAM family member and was found shortly following the completion of the Human Genome Project [90,98,99]. SLAMF9 has no ITSM similar to SLAMF8, but contains a large number of positively charged amino acids in its cytoplasmic domain [98]. SLAMF9 appears to be expressed on monocytes, pDCs, DCs, T cells, B cells, macrophages, and the monocytic cell line, THP-1 [98]. Of importance also, is that the ligand for SLAMF9 has yet to be identified. Studies of SLAMF9’s expression and function have been hampered by lack of good monoclonal antibodies against SLAMF9, particularly ones that are amenable to flow cytometry. 

While no direct role for SLAMF9 in anti-viral immunity has been discovered, roles for SLAMF9 on macrophages and pDCs have been described. A recent report identified SLAMF9 to be up-regulated on tumor-associated macrophages (TAMs) in melanoma [21]. Here, SLAMF9 was observed to enhance TNFα production from macrophages following LPS stimulation and was itself up-regulated following IFNγ stimulation [21]. The alteration in LPS-stimulated macrophage responses by SLAMF9 suggests that it may be important for anti-microbial immunity. Indeed, a published abstract has stated that SLAMF9 knockout mice display impaired clearance of *Salmonella Typhimurium* [100]. Additionally, it has been shown that SLAMF9 is highly expressed on pDCs, where it is able to regulate their function and mouse susceptibility to Experimental Autoimmune Encephalomyelitis (EAE) [29]. The ability of SLAMF9 to control pDCs function suggests that this receptor may play a role indirectly in controlling viral infections, as pDCs are known to be critical in this respect [101].

## 12. Potential for Targeting SLAM Family of Receptors in HIV and Other Viral Infections

Previously published reports by our group and others identified a critical role for SLAM family signaling in immune regulation suggesting the possibility of SLAM family modulation for therapeutic benefit during acute or chronic viral infections. While measles infections are rare in the United States due to the MMR vaccine, declining vaccination rates are making this infection more common, with potentially serious consequences for infected children. Monoclonal antibodies against SLAMF1, blocking the ability for measles to use this receptor as a cell entry mechanism, may prove to be a novel strategy to treat acute infection. A similar approach could be used against SLAMF3 to prevent its interactions with HCV. While blockade of SLAMF1 and SLAMF3 to directly prevent virus interactions are one approach, modulation of SLAMF9 signaling may allow for an indirect approach to treat acute or chronic viral infections. Monoclonal antibodies activating SLAMF9, specifically on pDCs, would, in theory, augment pDC IFN⍺ production and anti-viral responses [29]. While the approach may be beneficial during acute viral infections, an opposite approach may be useful in chronic HIV infection, where constitutive pDC activation is evident and pathogenic [36].

Targeted modulation of SLAMF7 signaling is additionally an exciting therapeutic approach in the setting of chronic HIV infection as we have shown that SLAMF7 signaling in human monocytes of healthy and HIV-infected patients counteracted type I interferon receptor-mediated signaling [18]. We also found that SLAMF7 signaling in human monocytes inhibits the production of various alpha chemokines, but not host restriction factors that are critical for combatting HIV-1 infection at the cellular level. These results have major implications, not only in HIV-associated pathogenesis, but also for other type I interferonopathies [102]. These discoveries suggest that SLAMF7 activation on monocytes of HIV infected individuals has the potential to prevent HIV infection of monocytes as well as pathogenic type I interferon signaling, all while sparing critical HIV host-restriction factors. 

Moreover, modulation of the SLAMF7-EAT-2 signaling pathway has the potential to be co-opted for enhanced vaccination responses. We have previously shown that blockade of SLAMF7 using a SLAMF7-Fc fusion protein augmented the development of HIV Gag-specific T cell immune responses following adenovirus-mediated vaccination [103]. Additionally, our previous work indicated that prolonged SLAM family signaling via EAT-2 over-expression augments vaccine-induced innate and adaptive immune responses [22,71,72,104,105]. 

Finally, some of the SLAM family members have recently been identified as T cell exhaustion markers [80,106], and signaling by these receptors impacts the development of proper T cell immune responses against cancer and viral antigens. Due to the huge success of CTLA-4 and PD-1 checkpoint blockade, it is tempting to speculate about the potential efficacy of SLAMF4 checkpoint blockade, as this receptor is a bona fide inhibitory receptor on CD8^+^ T cells. However, blocking SLAMF4 signaling on cytotoxic intra-tumoral T cells may be complicated by the fact that SLAMF4 actually seems to function as an activating receptor at low antigen densities [64]. 

## 13. Conclusions

Fueled by advances in multi-parametric flow cytometry and genomics, we now have the capability to rapidly discover and characterize new functions for the multitude of immune cell receptors. Of these immune receptors, the SLAM family represents a critical component in both the innate and adaptive arms of the immune system that requires further characterization. Their unique feature, self-ligation, makes them interesting targets, as their signaling is always triggered upon homotypic interactions between cells, or on the same cell (cis-interaction). Understanding their function in specific immune cells will facilitate the development of novel anti-viral and anti-cancer therapies. The conserved nature and ubiquitous functions of the SLAM family of receptors make them ideal targets for viruses looking to evade host immune responses. This has already been appreciated for a number of SLAM family receptors across a small number of viruses, but the true extent of SLAM receptor involvement remains to be seen. Studies of SLAM receptor signaling can be complicated by their homotypic interactions, differing roles across immune cell types, and occasional non-conserved functions between humans and mice. Additionally, co-expression studies and studies looking at combinatorial effects of SLAM family receptors have scarcely been performed. With the more wide-spread use of single-cell technologies we anticipate more detailed studies to reveal important complexities in SLAM signaling networks never before appreciated.

## Figures and Tables

**Table 1:** Signaling lymphocytic activation molecule (SLAM) family members and their known roles during viral infection. Cytomegalovirus, CMV; dendritic cells, DCs; hepatitis C virus, HCV; innate lymphoid cells, ILCs; hematopoietic stem cells, HLCs; NK cells, Natural Killer cells.

SLAM Family Member	Known Direct Interactions with A Virus	Known Immuno-Modulatory Roles during Viral Infection	Expression Pattern
SLAMF1 (SLAM, CD150)	Measles [39]	Th2 polarization [17,40]	T cells, B cells, macrophages, DCs, platelets, HSCs
SLAMF2 (CD48)	Down-regulated via CMV viral protein m154 [41]	Regulates DC survival and antigen-presentation during T cell interactions [31]	Nearly all hematopoietic cells
SLAMF3 (Ly-9, CD229)	HCV E2 protein [42]		CD4^+^ T cells, CD8^+^ T cells, B cells, plasma cells, NK cells, NKT cells, ILCs, DCs, monocytes, macrophages, and HSCs
SLAMF4 (2B4, CD244)	Can be blocked via interactions with CMV SLAMF2 decoy receptor [43] Influenza viral HA protein [44]	T cell exhaustion [45,46,47,48,49,50]	NK cells, CD8^+^ T cells, NKT cells, γδ T cells, monocytes, basophils, eosinophils, DCs, mast cells
SLAMF5 (CD84)		DC pro-inflammatory signaling [19] Germinal center formation [33]	B cells, T cells, monocytes, macrophages, DCs, platelets, thymocytes, NK cells, NKT cells, basophils, eosinophils.
SLAMF6 (NTB-A, Ly-108, CD352)	Degradation via interactions with HIV-1 vpu viral protein [51,52,53]	Cytotoxic interactions between virally infected cells and NK cells [44]	NK cells, NKT cells, T cells, B cells, macrophages, pDCs, DCs, thymocytes, eosinophils, neutrophils.
SLAMF7 (CRACC, CD319, CS1)		Regulates monocyte responses to type I interferons [18] Regulates monocyte susceptibility to HIV-1 infection [18]	NK cells, CD4^+^ T cells, CD8^+^ T cells, NKT cells, classical monocytes, inflammatory monocytes, macrophages, DCs, B cells, plasma cells
SLAMF8 (BLAME, CD353)		Regulation of macrophage pro- and anti-inflammatory functions [20]	B cells, T cells, monocytes, macrophages, DCs, platelets, thymocytes, NK cells, NKT cells, basophils, eosinophils.
SLAMF9 (SF2001, CD84H1)		Regulation of macrophage and pDC responses [21,29]	Monocytes, pDCs, DCs, T cells, B cells, macrophages.
